# De Novo Assembly of the Asian Citrus Psyllid *Diaphorina citri* (Hemiptera: Psyllidae) Transcriptome across Developmental Stages

**DOI:** 10.3390/ijms21144974

**Published:** 2020-07-14

**Authors:** Chunxiao Yang, Da Ou, Wei Guo, Jing Lü, Changfei Guo, Baoli Qiu, Huipeng Pan

**Affiliations:** 1 State Key Laboratory for Conservation and Utilization of Subtropical Agro-Bioresources, South China Agricultural University, Guangzhou 510642, China; yangchunxiao@scau.edu.cn; 2 Key Laboratory of Bio-Pesticide Innovation and Application of Guangdong Province, South China Agricultural University, Guangzhou 510642, China; ouda_IPM@stu.scau.edu.cn (D.O.); gwei8290@stu.scau.edu.cn (W.G.); 13556143025@stu.scau.edu.cn (J.L.); changfeiguo@163.com (C.G.); 3 State Key Laboratory for Biology of Plant Diseases and Insect Pests, Chinese Academy of Agricultural Sciences, Beijing 100081, China

**Keywords:** *Diaphorina citri*, transcriptome annotation, developmental stage, differential expression, trehalase

## Abstract

Asian citrus psyllid *Diaphorina citri* Kuwayama is an important economic pest of citrus, as it transmits *Candidatus* Liberibacter asiaticus, the causative agent of huanglongbing. In this study, we used RNA-seq to identify novel genes and provide the first high-resolution view of the of *D. citri* transcriptome throughout development. The transcriptomes of *D. citri* during eight developmental stages, including the egg, five instars, and male and female adults were sequenced. In total, 115 million clean reads were obtained and assembled into 354,726 unigenes with an average length of 925.65 bp and an N50 length of 1733 bp. Clusters of Orthologous Groups, Gene Ontology, and Kyoto Encyclopedia of Genes and Genomes analyses were conducted to functionally annotate the genes. Differential expression analysis highlighted developmental stage-specific expression patterns. Furthermore, two trehalase genes were characterized with lower expression in adults compared to that in the other stages. The RNA interference (RNAi)-mediated suppression of the two trehalase genes resulted in significantly high *D. citri* mortality. This study enriched the genomic information regarding *D. citri*. Importantly, these data represent the most comprehensive transcriptomic resource currently available for *D. citri* and will facilitate functional genomics studies of this notorious pest.

## 1. Introduction

The Asian citrus psyllid *Diaphorina citri* Kuwayama (Hemiptera: Psyllidae) is one of the most destructive pests of citrus, due primarily to the fact of its role as the vector of *Candidatus* Liberibacter asiaticus (*C*Las) which, worldwide, causes the highly destructive citrus disease huanglongbing (HLB) [1,2]. Huanglongbing is one of the most lethal diseases against citrus and, currently, there is no cure. *Diaphorina citri* was first described in Taiwan Province in 1907 [3], and the infectious nature of HLB was described in Southern China in 1956 [4]. Large populations of *D. citri* in HLB-endemic areas often result in high incidences and spread of HLB. Controlling *D. citri* to reduce its availability as a vector of CLas has been a pivotal global strategy to restrict the spread of HLB. The recent rapid spread of HLB in the Americas has motivated extensive research aimed at improving the understanding of *D. citri* biology, ecology, and management tactics [5].

Several factors contribute to the status of *D. citri* as a serious pest species, including adaptation to a wide range of host plants [6], a short life cycle and high fecundity [7], adaptation to growth at a wide range of temperatures [8,9], high dispersal ability [5], and high capacity to evolve resistance to different types of insecticides [5,10,11,12]. Intense use of insecticides has led to the development of high levels of insecticide resistance in *D. citri* populations [12]. Therefore, the development of novel highly specific and environmentally friendly methods must be considered for controlling this pest.

RNA interference (RNAi) technology is a powerful tool for functional studies that can be used to selectively suppress the expression of target genes both in vitro and in vivo [13,14,15,16,17,18]. RNA interference has been widely explored as an alternative to conventional management methods for controlling insect pests [15,16,17,18]. For example, bacterially expressed double-stranded RNA (dsRNA) from the *lesswright* (*lwr*) gene of *Henosepilachna vigintioctopunctata* was applied to eggplant leaves and caused 88% mortality for the 1st larvae of *H. vigintioctopunctata*, 66% mortality for the 3rd instar larvae, and 36% mortality to the adults after 10 d, 10 d, and 14 d of application, respectively [15].

The genome of *D. citri* has been sequenced in recent years (GCA_000475195.1). In addition, transcriptomes of the terminal abdomen and antennae of adult male and female *D. citri* (SRX1330478–SRX1330481), those of *C*Las-free and *C*Las-infected *D. citri* nymphs and adults (SRX525230, SRX525218, SRX525209, SRX525152), and those encompassing the three main life stages of *D. citri* (egg, nymph/larva, and adult) have also been sequenced [19]. *Diaphorina citri* undergoes gradual metamorphosis and, as noted, has three differentiated life stages. The metamorphic changes are accompanied by changes in gene expression. Molecular characterization of *D. citri* at its various stages of morphogenesis would likely provide important inroads into the identification of novel target sites for pest control. Furthermore, comparison of the developmental transcriptomes of *D. citri* from the eggs, each of the instars, and adult male and female insects would provide insights into the function of numerous genes and the regulation of different signaling pathways involved during the different developmental stages. 

Trehalose is a natural alpha-linked disaccharide formed by an α,α-1,1-glucoside bond between two α-glucose units. Trehalose can be used as an energy source by bacteria, fungi, insects, invertebrates, plants, and a large number of other organisms and plays an important role in stress tolerance [20]. Trehalose is hydrolyzed by the enzyme trehalase into glucose to rapidly provide energy when needed. Trehalase is also the first enzyme in the chitin biosynthesis pathway and significantly influences chitin metabolism by regulating the pathway. Therefore, trehalase has great potential as a target gene for controlling insects using RNAi technology [20,21,22].

In the current study, we attempted to obtain expression data throughout all developmental stages of *D. citri* by performing comprehensive RNA sequencing. In addition, two trehalase genes were characterized and their functions investigated using RNAi technology. The assembled and annotated transcriptome sequences and gene expression profiles should provide useful information for the identification of genes involved in *D. citri* development and will facilitate functional genomics studies on this destructive insect species.

## 2. Results

### 2.1. Illumina Sequencing and Assembly

**Figure 1:**
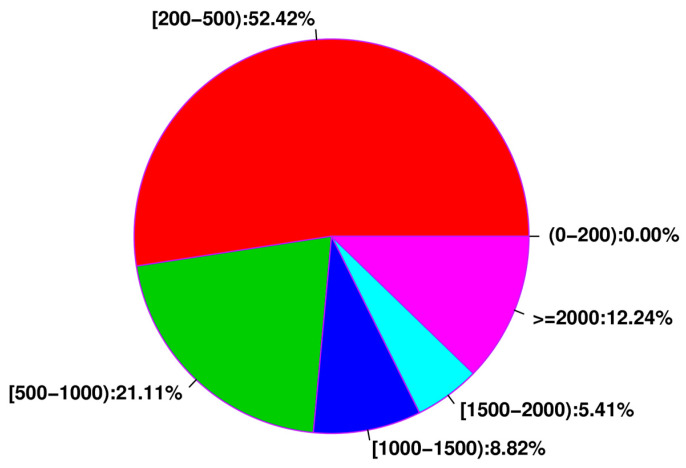
Unigene size distribution. Unigene sizes were calculated, and their relative distribution (%) according to size (bp) are shown.

To advance our understanding of the molecular mechanisms involved in the developmental changes that occur during *D. citri* life stages, complementary DNA (cDNA) libraries generated from eggs, 1st instars, 2nd instars, 3rd instars, 4th instars, 5th instars, and male and female adults were sequenced using the Illumina HiSeqTM 2000 sequencing platform. The results were deposited in the Sequence Read Archive (SRA) repository and are available using the following BioProject accession numbers: PRJNA633316, PRJNA634209, and PRJNA633417. The quality statistics of the filtered data are shown in Table S1. A total of 39,787,301 contigs were obtained with a mean length of 58.24 bp and an N50 length of 49 bp. After clustering the contigs with the nucleotide sequences available at the National Center for Biotechnology Information (NCBI), we ultimately obtained 354,726 unigenes with an average length of 925.65 bp and an N50 length of 1733 bp. The unigene length distribution indicated most of the unigenes (52.42%) were concentrated at 200–500 bp lengths (Figure 1).

### 2.2. Annotation of Predicted Proteins 

**Figure 2:**
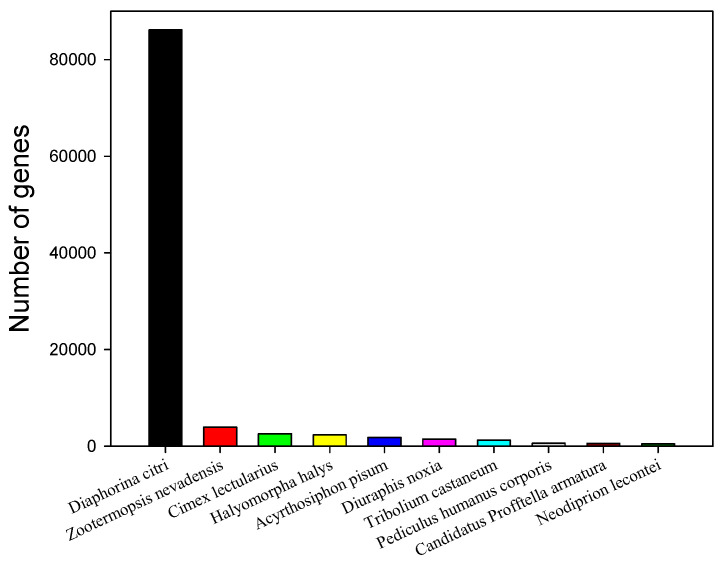
Top 10 species distribution of the BLASTX top hits results. Species distribution of the unigene BLASTX top hits results against the National Center for Biotechnology Information (NCBI) non-redundant protein database using a cutoff E-value of 10^−5^ and the proportions of each species are shown. Each bar represents a different species.

To annotate the unigenes, we first searched reference sequences using BLASTX against the non-redundant (NR) NCBI protein database using a cut-off E-value of 10^−5^. The annotation details for each unigene are provided in Table S2, with the matching protein listed was the top hit for each unigene (Table S2). A total of 120,902 of all the annotated unigenes (34.08%) provided a BLAST result. Gene number distribution among the best match top 10 species is shown in Figure 2. A total of 101,114 unigenes were annotated to the 10 top-hit species of insects of which 86,146 annotated genes (approximately 85.20%) were matched to *D. citri*, the number one top-hit species. The other top-hit species were *Zootermopsis nevadensis*, *Cimex lectularius*, *Halyomorpha halys*, *Acyrthosiphon pisum*, *Diuraphis noxia*, *Tribolium castaneum*, *Pediculus humanus corporis*, *Candidatus* Profftella armatura, and *Neodiprion lecontei* (Figure 2).

**Figure 3:**
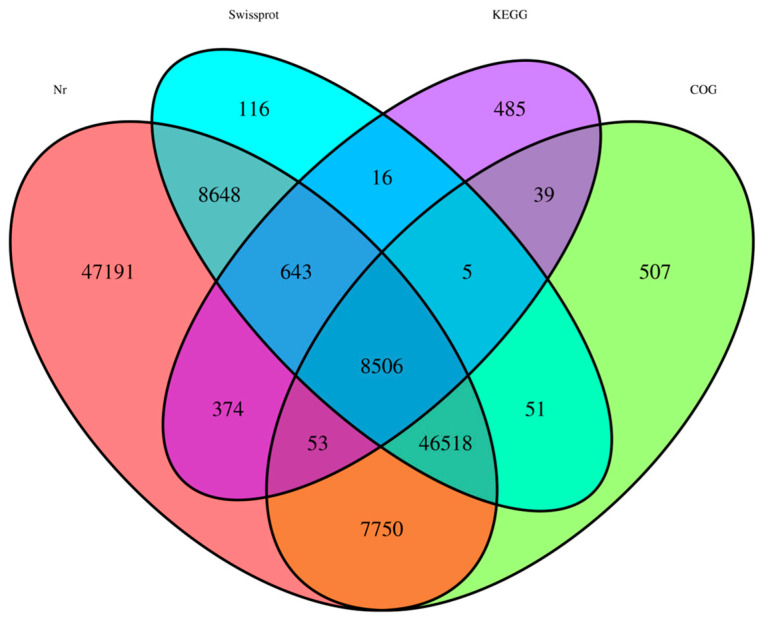
Comparison of unigene annotation in the National Center for Biotechnology Information (NCBI) non-redundant (Nr), SwissProt, Clusters of Orthologous Groups (COG), and Kyoto Encyclopedia of Genes and Genome (KEGG) databases.

A total of 120,902 unigenes were annotated based on four major databases, the NCBI Nr database, SwissProt database, Clusters of Orthologous Groups (COG) database, and Kyoto Encyclopedia of Genes and Genomes (KEGG) database (Table 1) (Figure 3). Among them, 119,683 unigenes were matched in the Nr database including 47,191 unigenes that matched only to this database. In addition, 64,503 unigenes had significant matches in the SwissProt database of which 116 unigenes only matched this database. Moreover, 63,429 unigenes had specific matches in the COG database and 10,121 unigenes had specific matches in the KEGG database, with 507 and 485 unigenes being unique to each database, respectively (Figure 3).

### 2.3. Functional Annotation Results 

**Figure 4:**
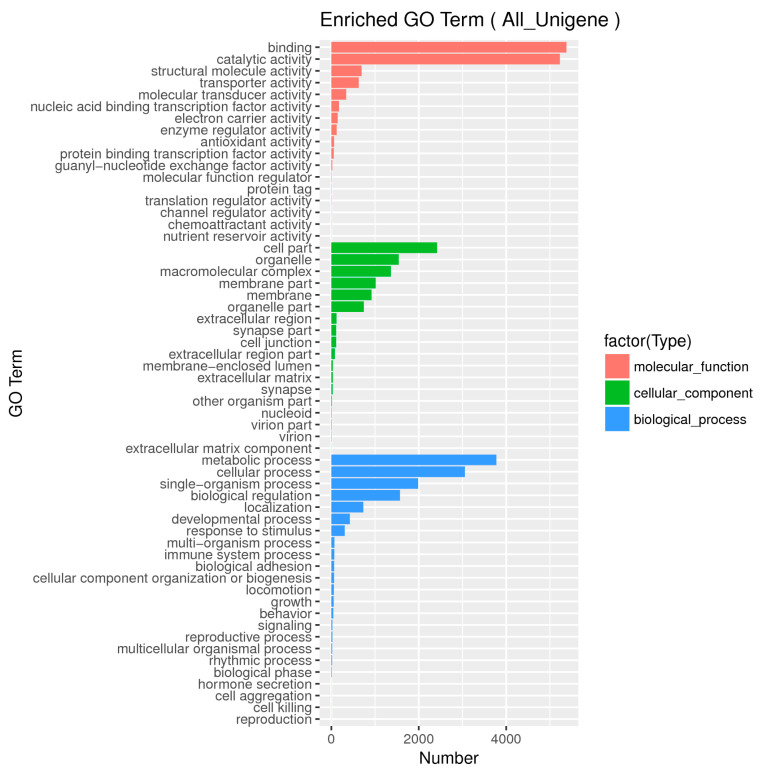
Gene Ontology (GO) functional categories of the unigenes annotated in the current study. Unigenes were annotated to three main categories: molecular function, cellular component, and biological process.

The functional classification of *D. citri* unigenes was predicted by performing Gene Ontology (GO), COG, and KEGG analyses. A total of 33,800 unigenes were assigned to three main categories of GO classification, molecular function (12,876 unigenes), cellular component (8548 unigenes), and biological process (12,376 unigenes). The terms “binding” and “catalytic activity”, “cell part” and “organelle”, and “metabolic process” and “cellular process” were the top two dominant GO terms for each of the three main categories (Figure 4). In total, 58 sub-categories were divided from the primary categories, 23 categories for “biological process,” 18 categories for “cellular component”, and 17 categories for “molecular function.”

**Figure 5:**
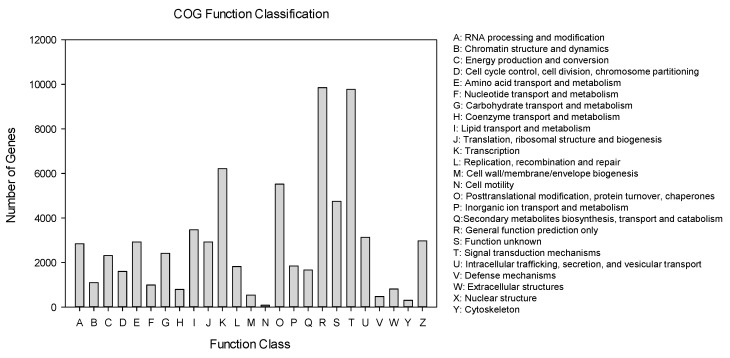
Clusters of Orthologous Groups (COG) functional categories of the unigenes annotated in the current study. The names of each class definition (A to Y) are provided on the right side of the figure.

For COG functional classification, a total of 71,145 unigenes were annotated to 25 COG categories (Figure 5). Among them, the largest group was the “general function prediction” (9856 genes, 13.85%), followed by the large groups (i.e., >2000 genes), “signal transduction mechanisms” (9776 genes, 13.74%), “transcription” (6216 genes, 8.74%), “posttranslational modification, protein turnover, chaperones” (5522 genes, 7.76%), and “lipid transport and metabolism” (3470 genes, 4.88%).

**Figure 6:**
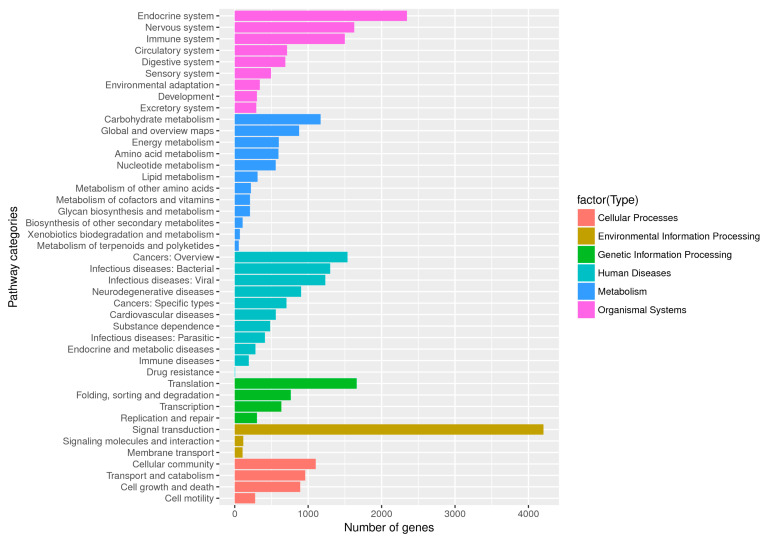
Kyoto Encyclopedia of Genes and Genome (KEGG) functional categories of the unigenes annotated in the current study. Unigenes were annotated and assigned to six main categories, cellular processes, environmental information processing, genetic information processing, human diseases, metabolism, and organismal systems.

*Diaphorina citri* unigene sequences that mapped to reference canonical pathways in the KEGG database were also analyzed. We specifically assigned 31,909 unigene sequences to 44 KEGG pathways. The top three pathways were signal transduction, endocrine system, and translation (Figure 6).

### 2.4. Differentially Expressed Genes (DEGs)

**Figure 7:**
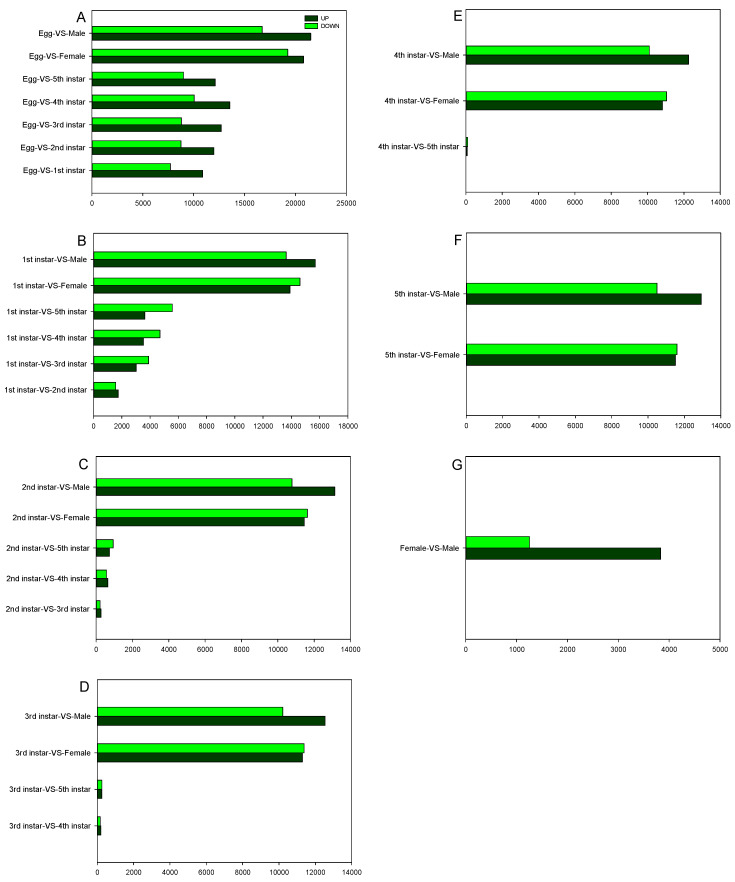
Comparison of differentially expressed gene (DEG) numbers in each. Upregulated (UP) and downregulated (DOWN) unigenes were quantified. The results of 28 comparisons are shown. (**A**) the comparison of DEGs between egg and other stages; (**B**) the comparison of DEGs between 1st instar and other stages; (**C**) the comparison of DEGs between 2nd instar and other stages; (**D**) the comparison of DEGs between 3rd instar and other stages; (**E**) the comparison of DEGs between 4th instar and other stages; (**F**) the comparison of DEGs between 5th instar and other stages; (**G**) the comparison of DEGs between female and male.

To identify DEGs among the different developmental stages, the number of clean tags for each gene was calculated. Gene expression variation was analyzed between different stage combinations (Figure 7). The top 20 upregulated and downregulated DEGs expressed genes at each stage are shown in Table S3.

### 2.5. Developmental Gene Expression of Trehalase 1 and Trehalase 2

**Figure 8:**
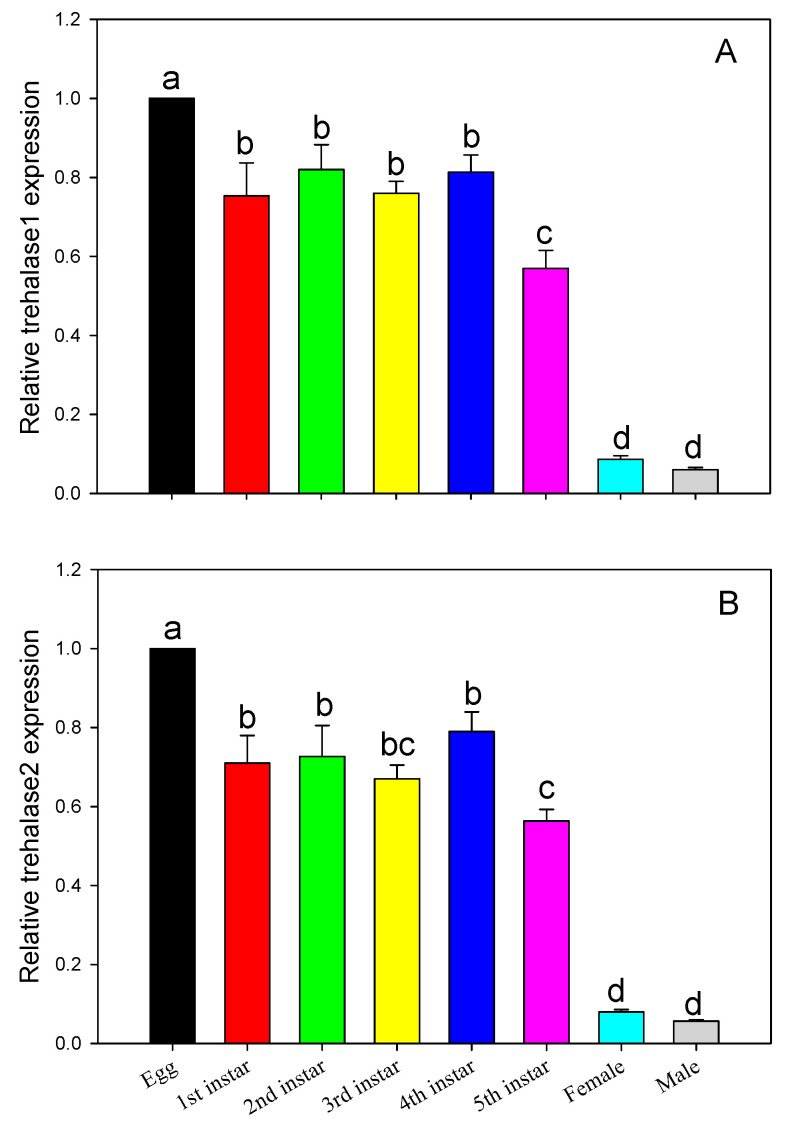
Expression patterns of trehalase 1 (**A**) and trehalase 2 (**B**) in *D. citri* across different developmental stages. The values shown are means + SE. Different letters indicate significant differences in gene expression across different developmental stages (*p* < 0.05).

Results from the gene expression analysis showed that trehalase 1 was differently expressed across different development stages (F_7,16_ = 62.363, *p* < 0.0001). Specifically, the trehalase 1 gene exhibited the highest expression level during the egg stage, while the expression level was lowest in the male and female adults. In addition, expression of trehalase 1 gene was lower in the 5th instar compared to that in the other four instar stages. There was no significant difference in trehalase 1 gene expression among the other development stages (Figure 8A). Similar gene expression profiles were found for the trehalase 2 gene across the different developmental stages (Figure 8B).

### 2.6. Impact of the silencing of trehalase 1 and trehalase 2 on Gene Expression

**Figure 9:**
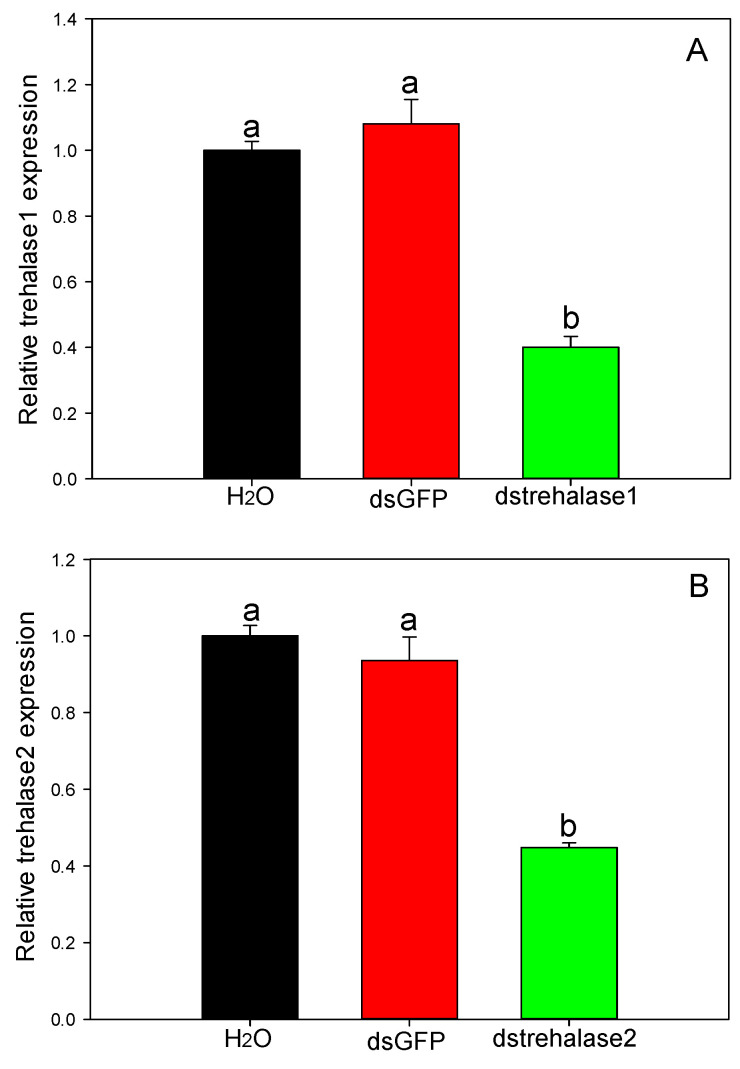
Relative gene expression in *D. citri* 2nd instars after treatment with specific double-stranded RNA (dsRNA). Trehalase 1 (**A**) and trehalase 2 (**B**). The 2nd instars were treated with trehalase1-specific dsRNA (dstrhalase1), trehalase2-specific dsRNA (dstrhalase2), control green fluorescent protein-specific dsRNA (dsGFP), or water (H_2_O) and the gene expression levels were measured. Values shown are means + SE. Different letters indicate significant differences among treatments (*p* < 0.05, Tukey’s multiple comparisons test).

After two days of treatment, gene expression of trehalase 1 in instars treated with trehalase1-specific dsRNA (dstrehalase1) was significantly suppressed (F_2,6_ = 77.681, *p* < 0.0001). The expression of trehalase 1 was reduced 2.495-fold compared to that of the ddH_2_O-treated control (Figure 9A). Similarly, gene expression of trehalase 2 in instars treated with trehalase2-specific dsRNA (dstrehalase2) was also significantly suppressed (F_2,6_ = 24.181, *p* = 0.001). The expression of trehalase 2 was reduced 2.232 fold compared to that of the ddH_2_O-treated control (Figure 9B).

### 2.7. Toxicity of in Vitro Synthesized Dstrehalase1 and Dstrehalase2

**Figure 10:**
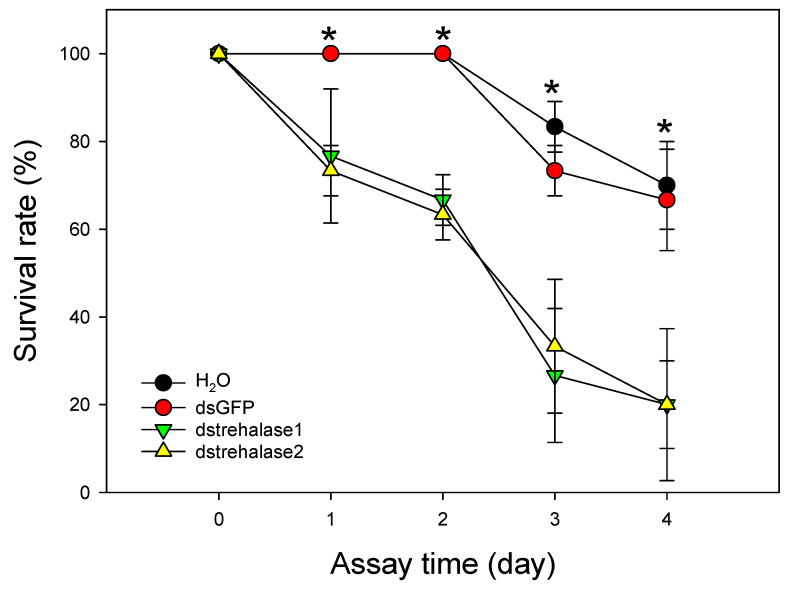
Effect of trehalase1-specific double-stranded RNA (dstrehalase1) and trehalase2-specific double-stranded RNA (dstrehalase2) treatment on *D. citri* 2nd instars mortality. *D. citri* 2nd instars were treated with dstrehalase1, dstrehalase2, green fluorescent protein-specific dsRNA (dsGFP), or water (H_2_O) and monitored for mortality. Values shown in the figure are means ± SE. * Indicates statistically significant differences (*p* < 0.05; ANOVA, Tukey’s multiple comparisons test).

*Diaphorina citri* mortality in the dstrehalase1- and dstrehalase2-treated groups was observed starting on the first day of dsRNA bioassay (Figure 10). Specifically, knockdown of trehalase 1 and trehalase 2 using their respective specific dsRNAs each resulted in significant mortality of *D. citri* compared to that in the dsGFP-treated and H_2_O-treated control groups on day 1 (F_3,8_ = 28.152, *p* < 0.0001), day 2 (F_3,8_ = 217.502, *p* < 0.0001), day 3 (F_3,8_ = 15.672, *p* = 0.001), and day 4 (F_3,8_ = 7.958, *p* = 0.009) post-treatment (Figure 10). There was no significant difference in *D. citri* mortality between the dstrehalase1-treated and dstrehalase2-treated groups at any time point.

## 3. Discussion

In the current study, we compared the transcriptomes of eight developmental stages of *D. citri*. A better understanding of the molecular mechanisms regulating the life cycles and developmental stages of this important pest may aid in its control by facilitating the development of more sustainable and environmentally friendly interventions. Our results significantly advance the molecular resources available for the study of this and other insect pests and provide a framework to understand changes in gene expression during insect development. We assembled transcriptomes from all eight developmental stages and identified a total of 354,726 unigenes with an average length of 925.65 bp. A total of 120,902 unigenes were annotated using four major databases.

Interestingly, transcriptome sequence analysis revealed that *Z. nevadensis*, which belong to the order Blattaria, shared the highest similarity with *D. citri* based on the BLAST annotation. This was in contrast to *H. halys*, *A. pisum*, and *D. noxia*, which like *D. citri* belong to the order Hemiptera but showed lower best-match percentages. These unexpected results were not likely due to the availability of more sequence resources for *Z. nevadensis* in the NCBI protein database compared to those for the other organisms, as the number of protein sequences for *A. pisum* (53,285) was higher than that for *Z. nevadensis* (49,273). It appears that *D. citri* may have a closer relationship to *Z. nevadensis* than to the other Hemipteran insects. The precise relationship among these insect species needs further investigation.

One of the top 10 species regarding distribution of the best match by BLASTX against the Nr database was Ca. Profftella armature. *D. citri* possesses a specialized organ called a bacteriome, which may harbor the vertically transmitted intracellular mutualists Ca. Carsonella ruddii and Ca. Profftella armature. Carsonella is a typical nutritional symbiont while Profftella is an unprecedented type of toxin-producing defensive symbiont, unusually sharing organelle-like features with nutritional symbionts [23,24]. Furthermore, many strains of *D. citri* are infected with *Wolbachia*, which manipulates the reproductive and developmental processes in various arthropod hosts [25]. In our study, Ca. Carsonella ruddii, Ca. Profftella armature, and *Wolbachia* were each found in the transcriptome, with Profftella having the most abundant transcripts (Table S2). The function of these symbionts in *D. citri* should be investigated in future studies.

Compared to that of the previous study in which the *D. citri* transcriptomes of eggs, nymphs, and adults were sequenced with no biological replicate [19], our transcriptome and gene expression profiling data greatly enrich the current *D. citri* transcriptome database and will benefit research with respect to the identification of novel genes, chemical targets, developmental mechanisms, and sex difference of *D. citri*. During the developmental stages, most DEGs were Upregulated and downregulated in adults compared to that in eggs. In contrast, the expression of few genes was changed among different larvae stages. Each stage library contained large numbers of genes that showed specific expression and were likely involved in developmental differentiation. This is consistent with other insects that also show their larvae stage development to be tightly regulated by a few key genes, and this regulation is important for developmental differentiation [26,27].

The success of RNAi-based strategies in future pest management will rely on the identification of essential target genes that confer lethal phenotypic effects upon knockdown [28]. Generally, functional genes involved in insect development, metamorphosis, or key metabolic processes may be suitable RNAi targets as part of control strategies of insects [15,16,17,18,29,30,31,32,33]. Previous studies have shown that trehalase plays pivotal roles in various physiological processes, including energy metabolism [34], chitin synthesis during molting [35], and diapause [36]. Recently, trehalases from several insects have been characterized and their biological roles investigated, including those from *Nilaparvata lugens* [21], *Tribolium castaneum* [22], and *Harmonia axyridis* [36]. Suppressing the expression of *trehalase* can lead to the developmental inhibition and death of the insects [21,22]. In the current study, treating *D. citri* nymphs with a solution containing dsRNAs specific for trehalase 1 and trehalase 2 genes resulted in a robust RNAi response. When dsRNA is administered by soaking the insect with the solution, the dsRNA can be taken up through spiracles or by cuticle permeation [37]. This is then followed by the spread of the dsRNA/siRNA molecules throughout the body of the insect. The individual insect cells can then take up the dsRNA/siRNA from their nearby environment, thereby inducing the RNAi activity, which is process referred to as environmental RNAi silencing [28]. Ultimately, we found that silencing trehalases induced high mortality in *D. citri*, which lays the foundation for developing RNAi-based biopesticides aimed at controlling *D. citri*.

In general, we developed a comprehensive sequence resource of *D. citri* consisting of a desirable quality. It was built based on preparing and analyzing eight different developmental transcriptomes of *D. citri* eggs, larvae, and adults. Future analyses of genes related to insect development or death will be useful for pest control. Furthermore, this study enriches the genomic platform of *D. citri* which should facilitate our understanding of the metamorphosis and development of this important pest insect.

## 4. Materials and Methods

### 4.1. Insect Rearing and Sample Preparation

*Diaphorina citri* adults were collected from *Murraya exotica* (L.) and reared on host *M. exotica* in an incubator at 25 ± 0.5 °C, a 16 h light/8 h dark photoperiod and 50% relative humidity. Eight developmental stages of *D. citri* were sampled, including eggs, 1st instars, 2nd instars, 3rd instars, 4th instars, 5th instars, and male and female adults. Each developmental sample was collected during the first two days of their respective stages. First to fifth instars were discriminated by appearance and body size. All the samples were placed in TRIzol reagent (Invitrogen, Carlsbad, CA, USA), rapidly frozen in liquid nitrogen, and then transferred to −80 °C. All *D. citri* developmental samples were represented by three biological replicates that were independently processed.

### 4.2. mRNA Sequencing by ILLUMINA HiSeq

Total RNA was extracted from the *D. citri* samples using an RNeasy Mini Kit (Qiagen, Hilden, Germany) according to the manufacturer’s instructions. After quantification of the RNA using an Agilent 2100 Bioanalyzer (Agilent Technologies, Santa Clara, CA, USA), a NanoDrop spectrophotometer (Thermo Fisher Scientific Inc., Waltham, MA, USA), and 1% agarose gel electrophoresis, the extracted RNA with RNA integrity number (RIN) values >8 was used for generation of cDNA libraries. Next generation sequencing libraries were constructed using a NEBNext^®^ Ultra™ RNA Library Prep Kit for Illumina^®^ according to the manufacturer’s protocol (New England Biolabs, Ipswich, MA, USA). Libraries with different indices were then multiplexed and loaded onto an Illumina HiSeqTM 2000 instrument according to manufacturer’s instructions (Illumina, San Diego, CA, USA). Sequencing was carried out using a 2 × 150 bp paired-end (PE) platform (GENEWIZ, Suzhou, China).

### 4.3. Functional Annotation Analysis of Sequencing Data

After sequencing, all sequence data were cleaned using Trimmomatic with default parameters [38]. Two approaches were then used for data analysis. For the first approach, expression profiles of each gene at different developmental stages were determined. During this step, both assembly and annotations of *D. citri* were downloaded from NCBI and used as references. Briefly, the clean data were mapped to the *D. citri* genome using Hisat2 [39]. Gene values, represented as transcripts per kilobase million (TPM), were then calculated using StringTie [40]. Differentially expressed genes between every two samples were also determined using DESeq with a cutoff for fold change set at > 2 and false discovery rate (FDR) < 0.05 [41]. For the second approach, a transcript dataset of *D. citri* was constructed. Briefly, all the clean reads were assembled using Trinity [42] and the transcripts generated were clustered by CD-Hit software [43]. Ultimately, transcript datasets of *D. citri* were constructed using the above approaches. A database consisting of NR, SwissProt, COG, GO, and KEGG gene sequences and related parameters were analyzed using the appropriate software (Table S4).

### 4.4. Temporal Gene Expression Analysis of Trehalase

RNAs from different developmental stages used for Illumina HiSeq sequencing were used for gene expression analysis. First-strand cDNA was prepared using 1 μg of total RNA and a PrimeScript RT Kit with gDNA Eraser (Perfect Real Time) from TaKaRa (Dalian, China) following the manufacturer’s instructions. The synthesized first-strand cDNA was diluted 10 fold and was immediately used or stored at −20 °C until used.

Real-time reverse transcription polymerase chain reaction (RT-qPCR) was conducted according to the manufacturer’s recommendations using SYBR^®^ Premix Ex Taq™ (Tli RNaseH Plus) purchased from TaKaRa. PCR amplification in 15 μL reactions were performed using the cycling program and PCR system described in our previous study [44]. Briefly, PCR reaction mixtures contained 5.25 μL ddH_2_O, 7.5 μL 2× SYBR Green MasterMix (Bio-Rad Laboratories, Hercules, CA, USA), 4 μM each specific primer, and 1.0 μL first-strand cDNA template. The RT-qPCR program included an initial denaturation at 95 °C for 3 min followed by 40 cycles of denaturation at 95 °C for 10 s, annealing for 30 s at 55 °C, and extension for 30 s at 72 °C. The gene-specific primers used for the RT-qPCR are listed in Table 2. Relative quantification was calculated using the 2^−ΔΔCt^ method [45] and data were normalized to expression of reference genes *GAPDH* and *EF1α* [46]. Three technical replicates were used for each sample.

### 4.5. dsRNA Synthesis

Specific dsRNA primers containing a T7 promoter sequence at the 5′end targeting trehalase 1 and trehalase 2 were designed using E-RNAi. The sequences of the primers are listed in Table 3. The PCR template and cycling program used were described in our previous study [44]. PCR products were purified using a Universal DNA Purification Kit (TIANGEN, Beijing, China) and used as template with a T7 MEGAscript Kit (Thermo Fisher Scientific) following the manufacturer’s protocol to generate the dsRNA. The primers for dsGFP synthesis were used according to our recent study [16]. Synthesized dsRNAs were purified and suspended in ddH_2_O and their size and integrity confirmed by 1.5% agarose/TAE gel electrophoresis stained with *GoldView I*. Concentrations of the purified dsRNAs were determined using a NanoDrop One spectrophotometer (Thermo Fisher Scientific), and the purified dsRNAs were stored at −20 °C until use.

### 4.6. RNA Interference Assays on D. citri

In our preliminary investigation, the effect of dsRNA on *D. citri* 2nd instars mortality was determined using dstrehalase concentrations of 10, 25, 50, 75, and 100 ng/μL. We found that *D. citri* 2nd instars had the highest mortality rates when exposed to 75 ng/μL dstrehalase and the mortality did not increase when the dstrehalase concentration was increased to 100 ng/μL. Therefore, the concentration of dsRNA used in this study was 75 ng/μL. RNA interference assays were conducted according to a recent study [14] with modification. Specifically, *D. citri* 2nd instars were starved for two hours and then completely soaked with a 4 μL droplet containing 75 ng/μL dsRNA. The dsRNA droplet was removed using a pipette after soaking for approximately 2 min. As controls, additional *D. citri* 2nd instars were treated with ddH_2_O or dsGFP. After drying on filter paper, 20 newly emerged *D. citri* 2nd instars were transferred onto 5 to 7 *M. paniculata* seedlings of a true leaf stage (tender leaf). The petiole was then fixed in agar at the bottom of a glass cylindrical container (4 cm diameter and 20 cm high), the upper opening of the cylinder was covered with a mesh screen for ventilation, and the agar was covered with a piece of filter paper and cotton to prevent water loss. The feeding chambers were placed incubated at 25 ± 0.5 °C with a 16 h light/8 h dark photoperiod and 50% relative humidity. Each treatment was performed using three biological replicates. Mortality was monitored and recorded for four days.

For analysis of gene suppression caused by the cognate mRNAs, *D. citri* 2nd instars were treated as noted above. A total of 100 instars were used for each treatment. After two days, 50 living nymphs were collected, flash-frozen in liquid nitrogen, and stored at –80 °C in 1.5 mL centrifuge tubes until processed for total RNA extraction. Each treatment was independently repeated three times. For RT-qPCR analysis, total RNA was extracted, cDNA synthesized, and RT-qPCR amplification performed as described above. Three technical replicates were used for each sample.

### 4.7. Data Analysis

One-way analysis of variance (ANOVA) was used to detect significant differences in trehalase 1 and trehalase 2 expression levels among different treatments, followed by a Tukey’s multiple range test with *p* < 0.05 being considered statistically significant. One-way ANOVA was also used to compare mortality rates each day of *D. citri* treated with H_2_O, dsGFP, dstrehalase1, and dstrehalase2. Mortality means were compared using Tukey’s tests and again *p* < 0.05 was considered statistically significant. Proportional data were arcsine square root transformed before analyses. All statistical analyses were performed using SPSS version 21.0 software (SPSS Inc., Chicago, IL, USA).

## 5. Conclusions

Our data greatly improved our genetic understanding of *D. citri* and provides a large number of gene sequences for future studies. Our findings also provide comprehensive insight into gene expression profiles across different developmental stages of *D. citri*. Dietary RNAi toxicity assays demonstrated that trehalase 1 and trehalase 2 may be effective molecular targets for controlling *D. citri*. However, prior to the practical application of RNAi to control *D. citri*, further studies are necessary, such as determining an effective delivery system of dsRNA to citrus trees.

## Figures and Tables

**Table 1:** Statistics of annotation results.

Database	Nr	SwissProt	COG	KEGG	Total
Unigenes	119,683	64,503	63,429	10,121	120,902

**Table 2:** Assembly statistics for *Diaphorina citri* transcriptomes across developmental stages.

Type	Total Sequences	GC Percentage	N50	Max Length	Min Length	Average Length	Total Assembled Bases
Contig	39,787,301	42.54%	49	19,067	25	58.24	2,317,181,757
Unigene	354,726	39.43%	1733	19,592	201	925.65	328,351,381

**Table 3:** Primers used in this study.

Name of Primers	Primer Sequences (5′-3′)
dstrehalase1-F	TAATACGACTCACTATAGGGGGGCGTGATCGAGAACATAA
dstrehalase1-R	TAATACGACTCACTATAGGGGATGAACCACCGACTGGAAA
dstrehalase2-F	TAATACGACTCACTATAGGGCTTTGAAGCAGGCAATGAGC
dstrehalase2 -R	TAATACGACTCACTATAGGGGCACAATCTGTTGCCGATTT
RT-qPCR-trehalase1-F	TTTCCAGTCGGTGGTTCATC
RT-qPCR-trehalase1-R	CCACCATTCGCTCAGATAGTT
RT-qPCR-trehalase2-F	CGAGCCTTGCTCACCAATAA
RT-qPCR-trehalase2-R	CTCGGGTTGGAGGTGAAATC

## Supplementary Materials

Supplementary materials can be found at https://www.mdpi.com/1422-0067/21/14/4974/s1. Table S1. The quality statistics of the filtered sequencing data. Table S2. The Nr annotation information for each unigene. Table S3. The top 20 upregulated and downregulated differential expression genes expressed genes at each stage. The stage on the right for each DEG comparison served as the control. Table S4. Gene function analysis software and parameters.

## Abbreviations

Nr	Non-redundant
COG	Clusters of Orthologous Groups of proteins
GO	Gene Ontology
KEGG	Kyoto Encyclopedia of Genes and Genomes
RT-qPCR	reverse transcriptase-quantitative polymerase chain reaction
