# Supplementary material for: De Novo Assembly of the Asian Citrus Psyllid Diaphorina citri (Hemiptera: Psyllidae) Transcriptome across Developmental Stages
Source: Int J Mol Sci. 2020 Jul 14;21(14):4974. doi: 10.3390/ijms21144974 (PMC7404022; doi:10.3390/ijms21144974)
Supplement: Supplementary file 1 [file ijms-21-04974-s001.zip › Table S4.docx]

**Table S4**. Gene function analysis software and parameters

| **Sequence** | **Software** | **Format** | **Parameter** |
| --- | --- | --- | --- |
| NR | DIAMOND | v0.8.37.99 | 1e-5 |
| Swiss-Prot | DIAMOND | v0.8.37.99 | 1e-5 |
| Pfam | HMMER3 | 3.1b2 | default parameters |
| COG | DIAMOND | v0.8.37.99 | 1e-5 |
| GO | BLAST2GO | 2.5.0 | default parameters |
| KEGG | KOBAS | 2.1.1 | default parameters |
