# Supplementary material for: De Novo Assembly of the Asian Citrus Psyllid Diaphorina citri (Hemiptera: Psyllidae) Transcriptome across Developmental Stages
Source: Int J Mol Sci. 2020 Jul 14;21(14):4974. doi: 10.3390/ijms21144974 (PMC7404022; doi:10.3390/ijms21144974)
Supplement: Supplementary file 1 [file ijms-21-04974-s001.zip › Table S1.docx]

**Table S1**. The quality statistics of the filtered sequencing data

| Sample | Length | Reads | Bases | Q20 (%) | Q30 (%) | GC (%) |
| --- | --- | --- | --- | --- | --- | --- |
| Eggs-1 | 148.88 | 43292450 | 6445316556 | 97.42 | 93.58 | 41.1 |
| Eggs-2 | 148.94 | 42576548 | 6341191944 | 97.49 | 93.72 | 41.22 |
| Eggs-3 | 148.91 | 41412218 | 6166670231 | 97.44 | 93.6 | 41.54 |
| 1st instar-1 | 148.87 | 39563534 | 5889758912 | 97.61 | 94.01 | 40.49 |
| 1st instar-2 | 148.79 | 41497526 | 6174258122 | 97.58 | 93.93 | 41.02 |
| 1st instar-3 | 148.73 | 39655970 | 5898102829 | 97.55 | 93.85 | 41.13 |
| 2nd instar-1 | 148.07 | 46900212 | 6944334771 | 96.32 | 90.7 | 42.1 |
| 2nd instar-2 | 147.96 | 46738770 | 6915236286 | 96.53 | 91.13 | 42.16 |
| 2nd instar-3 | 147.92 | 53768750 | 7953522818 | 96.55 | 91.19 | 41.62 |
| 3rd instar-1 | 147.88 | 50679512 | 7494421474 | 96.35 | 90.8 | 42.34 |
| 3rd instar-2 | 147.99 | 54041490 | 7997683181 | 96.35 | 90.76 | 42.08 |
| 3rd instar-3 | 147.94 | 48597528 | 7189682589 | 96.37 | 90.82 | 41.5 |
| 4th instar-1 | 148.08 | 52050926 | 7707707064 | 96.37 | 90.8 | 42.13 |
| 4th instar-2 | 148.1 | 46539112 | 6892535837 | 96.42 | 90.9 | 41.62 |
| 4th instar-3 | 148.05 | 50076122 | 7413634147 | 96.27 | 90.58 | 42.34 |
| 5th instar-1 | 148.02 | 49409184 | 7313356676 | 96.39 | 90.83 | 41.92 |
| 5th instar-2 | 148.16 | 50698678 | 7511296957 | 96.36 | 90.77 | 41.99 |
| 5th instar-3 | 148.04 | 50978746 | 7547054203 | 96.31 | 90.65 | 42.33 |
| Female-1 | 148.27 | 50837888 | 7537905455 | 96.57 | 91.32 | 39.1 |
| Female-2 | 148.22 | 51043612 | 7565819293 | 96.57 | 91.3 | 39.83 |
| Female-3 | 148.38 | 50293172 | 7462600250 | 96.63 | 91.45 | 39.03 |
| Male-1 | 148.14 | 49197026 | 7287992271 | 96.28 | 90.65 | 39.9 |
| Male-2 | 148.27 | 53652954 | 7955386206 | 96.46 | 91.03 | 40.46 |
| Male-3 | 148.28 | 46982052 | 6966503192 | 96.57 | 91.29 | 39.76 |
